# Moderate-to-vigorous Physical Activity and Total and Site-specific Cancer: The Japan Public Health Center-based Prospective Study

**DOI:** 10.2188/jea.JE20250041

**Published:** 2026-03-05

**Authors:** Takashi Matsunaga, Hiroyuki Kikuchi, Shigeru Inoue, Hikaru Ihira, Taiki Yamaji, Motoki Iwasaki, Manami Inoue, Shoichiro Tsugane, Norie Sawada

**Affiliations:** 1 Division of Cohort Research, National Cancer Center Institute for Cancer Control, Tokyo, Japan; 2 Department of Preventive Medicine and Public Health, Tokyo Medical University, Tokyo, Japan; 3 Department of Physical Therapy, School of Health Sciences, Sapporo Medical University, Sapporo, Japan; 4 Division of Epidemiology, National Cancer Center Institute for Cancer Control, Tokyo, Japan; 5 Division of Prevention, National Cancer Center Institute for Cancer Control, Tokyo, Japan; 6 Graduate School of Public Health, International University of Health and Welfare, Tokyo, Japan

**Keywords:** Japanese community-dwelling adults, moderate-to-vigorous physical activity, prospective cohort study, site-specific cancer, total cancer

## Abstract

**Background:**

A World Health Organization guideline recommends that adults engage in moderate-to-vigorous physical activity (MVPA). We aimed to clarify associations of total MVPA in any domain with overall and site-specific cancer incidence using Japanese population-based cohort data.

**Methods:**

This study evaluated 84,054 participants (39,053 males and 45,001 females aged 50–79 years) of the 10-year survey of the Japan Public Health Center-based Prospective Study over a median follow-up of 13.0 years. Total MVPA was calculated based on a self-reported physical questionnaire, and 7.5 metabolic equivalent-hours/week (MET-hours/week) was defined as the minimum amount recommended by the guideline. Associations of categorized total MVPA with overall and site-specific cancer incidences were examined using multivariable-adjusted Cox proportional hazard models by sex.

**Results:**

Among males, the risk reduction was non-significant even in the highest MVPA category compared with no total MVPA. Among females, in contrast, risk of total cancer was reduced even when they engaged in lower total MVPA than the recommended amount (0.1–7.4 MET-hours/week) compared with no total MVPA (hazard ratio 0.79; 95% confidence interval, 0.65–0.97), and no further risk reduction was observed with increasing MVPA. Regarding site-specific cancers, engaging in higher total MVPA was inversely associated with risks of colon cancer (males) and bladder and endometrial cancers (females).

**Conclusion:**

Total MVPA was associated with reduced risk of overall cancer incidence in females, but not in males.

## INTRODUCTION

Regular physical activity has been linked to reduced incidence and mortality risks of total and several site-specific cancers.^1^ A World Health Organization (WHO) 2020 guideline recommends that adults engage in at least 150–300 minutes/week of moderate-intensity aerobic physical activity (MPA); at least 75–150 minutes/week of vigorous-intensity aerobic physical activity (VPA); or an equivalent combination of these two activities.^2^ A recent meta-analysis of 126 cohort studies^3^ and a pooled analysis of nine cohort studies^4^ have shown that an amount of leisure-time physical activity equal to that of the WHO recommendation decreases the incidence risks of total and some site-specific cancers.

However, some knowledge gaps exist with regard to physical activity recommendations for cancer prevention. First, although many previous studies focused on leisure-time physical activity,^5^ which accounts for only a small amount of the total activity people engage in daily,^6^ the amount of physical activity recommended by WHO counts physical activity in any of four domains: leisure-time, occupation, household, and transportation.^2^ Moreover, an umbrella review of systematic reviews has reported beneficial effects of occupational physical activity on site-specific cancers which have hitherto been considered harmful.^7^ Accordingly, current evidence for cancer prevention, mainly based on leisure-time physical activity, should be reviewed. Second, the amount of activity recommended by the WHO considers only moderate-to-vigorous physical activity (MVPA).^2^ To our knowledge, however, few studies have examined the association between total MVPA and overall cancer incidence, and whether the recommended amount can reduce total cancer incidence remains uncertain.^1^ Third, evidence for associations between physical activity and some site-specific cancers is inconsistent,^1^ and examining these associations would provide insights into research and clinical practice.

Here, we aimed to clarify associations of total MVPA with overall cancer (our primary outcome) and site-specific cancer incidence (our secondary outcome) using Japanese population-based cohort data.

## METHODS

### Study design and participants

The Japan Public Health Center-based Prospective Study (JPHC study) is a nationwide population-based cohort study launched in 1990–1994. Details of the cohort have been reported elsewhere.^8^ The baseline survey enrolled residents aged 40–69 years in 11 public health center areas. Participants were informed of the study’s objectives, and those who completed a questionnaire were regarded as consenting to participate. The 5-year and 10-year surveys were conducted to obtain updated information on lifestyle, including physical activity and health status. The present study regarded the 10-year survey as the starting point because the 10-year survey collected information necessary to calculate the amount of total MVPA, and its physical activity questionnaire was validated.

At the baseline survey conducted in 1990–1994, 140,420 individuals were identified as potential participants. Among these, the present study excluded 278 individuals who did not meet the eligibility criteria (non-Japanese nationality [*N* = 52], late report of emigration occurring before the follow-up period [*N* = 207], incorrect birth date [*N* = 7], or duplicate enrollment [*N* = 12]), 7,078 participants from the Tokyo area who lacked data on cancer incidence, and 20,563 participants who were censored before the 10-year follow-up survey. Among potential participants of the 10-year follow-up survey (*N* = 112,501), 93,687 responded to the 10-year follow-up survey, which was conducted between 2000–2003 (response rate: 83.3%). The present study further excluded 2,841 participants who had a physical disability, 1,664 who were missing data necessary to calculate physical activity, 4,112 who had a history of cancer, and 1,016 who had cancer diagnosis before the 10-year follow-up survey. Finally, 84,054 participants were eligible for the present analysis (eFigure 1).

### Exposures

The physical activity questionnaire of the present study consisted of items concerning leisure-time and non-leisure-time physical activity (eMaterial 1).^9^ The items on leisure-time physical activity asked participants about the frequencies and duration of the following activities during the previous year: 1) strolling, 2) brisk walking, 3) moderate intensity tasks such as playing golf and gardening, and 4) vigorous intensity tasks such as jogging, aerobics, and swimming. The frequency categories (five levels) ranged from <1 time/month to almost daily, and the duration categories (six levels) from <30 minutes to ≥4 hours. The items of non-leisure-time physical activity (occupational, household, and transportation activities) asked participants about the duration of the following tasks during non-leisure time on a typical day in the previous year: 1) sitting, 2) standing, 3) walking, and 4) vigorous tasks. The duration categories (eight levels) ranged from none to ≥11 hours. The following intensities (metabolic equivalents of task [METs]) were assigned to each task: 2.8 for strolling, 4.0 for brisk walking, 3.0 for moderate intensity tasks, 6.0 for vigorous intensity tasks, 1.3 for sitting, 2.0 for standing, 3.0 for walking, and 6.0 for vigorous tasks. For the frequency and duration of each task, midpoints of their categories were assigned to calculate MVPA. Amounts of leisure-time and non-leisure-time MVPA (MET-hours/week) were calculated by adding products of intensity, frequency, and duration for each task that had an intensity of 3.0 METs or higher, and total MVPA was calculated as the sum of leisure-time and non-leisure-time MVPAs. Our previous validation study comparing the physical activity questionnaire with 24-hour activity records showed acceptable reliability and validity for total MVPA (*r* = 0.645 and 0.610, respectively).^9^

### Covariates

When examining the association between MVPA and total cancer incidence, the following demographic, lifestyle, and comorbidity factors were considered as covariates: age (continuous), sex, residential area (10 study areas), occupation, smoking status, alcohol consumption, fruit and vegetable intake, sitting time, body mass index, diabetes, age at menopause (for females only), and use of hormone replacement therapy (for females only). These variables are considered cancer risk factors.^10^ Regarding site-specific cancers, we selected covariates by creating directed acyclic graphs based on the literature (eg, salted food intake for gastric cancer) (eFigure 2).^11^ These covariates are generally consistent with previous studies included in meta-analyses of site-specific cancers.^12^^–^^21^ Covariates other than age, sex, and residential area were created based on self-reported information gathered using the 10-year survey questionnaire. Continuous variables other than age were categorized to address nonlinear relationships. The cutoff values of categorical variables are shown in Table 1 and Table 2.

**Table 1.** Characteristics of participants according to total MVPA among males

Characteristics	Total MVPA (MET-h/wk)^a^				

	Not meeting the guideline’s recommendation		Meeting the guideline’s recommendation		
	0	0.1–7.4	T1 (7.5–62.9)	T2 (63.0–170.5)	T3 (≥170.6)
Number of participants	3,474	1,539	11,333	11,368	11,339
Total MVPA, MET-h/week, median (IQR)	0	1.0 (0.8–3.4)	31.5 (15.6–41.6)	101.9 (78.8–128.5)	265.4 (213.8–331.3)
Non-leisure-time MVPA, MET-h/week, median (IQR)	0	0.0 (0.0–0.0)	31.5 (10.5–31.5)	94.5 (63.0–126.0)	260.4 (210.0–324.0)
Leisure-time MVPA, MET-h/week, median (IQR)	0	1.0 (0.8–3.4)	2.9 (0.8–11.3)	2.3 (0.0–15.8)	0.8 (0.0–7.4)
Age, years, mean (SD)	65.9 (7.9)	63.1 (7.6)	61.1 (7.7)	61.3 (7.4)	60.8 (7.0)
Smoking status, pack-years, *n* (%)					
Never smoker	939 (27.0)	385 (25.0)	3,075 (27.1)	3,283 (28.9)	3,510 (31.0)
Past smoker	998 (28.7)	479 (31.1)	3,676 (32.4)	3,470 (30.5)	2,872 (25.3)
0.1–30.0 pack-years	378 (10.9)	149 (9.7)	1,024 (9.0)	1,041 (9.2)	1,155 (10.2)
>30.0 pack-years	972 (28.0)	491 (31.9)	3,394 (30.0)	3,402 (29.9)	3,615 (31.9)
Missing	187 (5.4)	35 (2.3)	164 (1.5)	172 (1.5)	187 (1.7)
Alcohol consumption, g/week, *n* (%)					
Nondrinker	1,405 (40.4)	569 (37.0)	3,172 (28.0)	2,965 (26.1)	2,951 (26.0)
Occasional drinker	195 (5.6)	117 (7.6)	945 (8.3)	884 (7.7)	814 (7.2)
<150 g/week	395 (11.4)	158 (10.3)	1,498 (13.2)	1,440 (12.7)	1,279 (11.3)
150–299 g/week	374 (10.8)	199 (12.9)	1,532 (13.5)	1,488 (13.1)	1,378 (12.2)
300–449 g/week	334 (9.6)	160 (10.4)	1,305 (11.5)	1,481 (13.0)	1,512 (13.3)
≥450 g/week	558 (16.1)	307 (20.0)	2,736 (24.1)	2,937 (25.8)	3,217 (28.4)
Missing	213 (6.1)	29 (1.9)	145 (1.3)	173 (1.5)	188 (1.7)
Body mass index, kg/m^2^, *n* (%)					
<18.5	157 (4.5)	53 (3.4)	295 (2.6)	270 (2.4)	296 (2.6)
18.5–24.9	2,083 (60.0)	974 (63.3)	7,250 (64.0)	7,482 (65.8)	7,666 (67.6)
25.0–29.9	856 (24.6)	428 (27.8)	3,347 (29.5)	3,191 (28.1)	2,928 (25.8)
≥30.0	92 (2.7)	50 (3.3)	308 (2.7)	285 (2.5)	231 (2.0)
Missing	286 (8.2)	34 (2.2)	133 (1.2)	140 (1.2)	218 (2.0)
History of diabetes, *n* (%)					
No	3,116 (90.0)	1,378 (89.5)	10,178 (89.8)	10,393 (91.4)	10,501 (92.6)
Yes	358 (10.3)	161 (10.5)	1,155 (10.2)	975 (8.6)	838 (7.4)
Occupation, *n* (%)					
Agriculture/forestry/fishery	761 (21.9)	177 (11.5)	1,140 (10.1)	2,697 (23.7)	3,880 (34.2)
Hired/self-employed/professional/others	962 (27.7)	657 (42.7)	7,107 (62.7)	6,078 (53.5)	5,430 (47.9)
Non-employed/unemployed	1,002 (28.8)	506 (32.9)	2,221 (19.6)	1,482 (13.0)	499 (4.4)
Missing	749 (21.6)	199 (12.9)	865 (7.6)	1,111 (9.8)	1,530 (13.5)
Sitting time, hours/day, *n* (%)					
<1.0	715 (20.6)	503 (32.7)	2,616 (23.1)	2,844 (25.0)	4,306 (38.0)
1.0–2.9	148 (4.3)	93 (6.0)	1,958 (17.3)	3,371 (29.7)	3,166 (27.9)
3.0–4.9	137 (3.9)	75 (4.9)	1,885 (16.6)	2,116 (18.6)	1,255 (11.1)
5.0–6.9	150 (4.3)	89 (5.8)	1,836 (16.2)	1,175 (10.3)	640 (5.6)
≥7.0	286 (8.2)	265 (17.2)	2,239 (19.8)	1,111 (9.8)	1,081 (9.5)
Missing	2,038 (58.7)	514 (33.4)	799 (7.1)	751 (6.6)	891 (7.9)
Food group intakes					
Fruits and vegetable intake, g/day, median (IQR)^b^	281.8 (152.9–457.9)	294.8 (174.6–459.8)	318.1 (198.8–474.1)	316.8 (198.9–473.7)	295.0 (183.7–446.1)
Salted foods intake, g/day, median (IQR)^b^	6.9 (1.0–20.6)	9.6 (1.7–22.3)	9.4 (1.7–20.5)	9.5 (1.9–19.6)	9.9 (1.8–21.1)
Red and processed meat intake, g/day, median (IQR)^b^	30.8 (10.2–62.2)	33.3 (15.0–58.6)	34.2 (17.2–57.5)	33.3 (16.7–56.0)	32.4 (15.9–55.7)

IQR, interquartile range; MET-h/wk, metabolic equivalent hours per week; MVPA, moderate-vigorous physical activity; SD, standard deviation; T, tertile.

^a^Cut-off values of tertiles were calculated only among participants with a total MVPA of 7.5 MET-h/wk or higher.

^b^Food group intakes were energy-adjusted using the residual method and incorporated as categorical variables into regression models: fruits and vegetable <189.9, 189.9–306.7, 306.8–464.7, and ≥464.8 g/day; salted foods <1.6, 1.6–9.3, 9.4–20.4, and ≥20.5; red and processed meat <16.1, 16.1–33.0, 33.1–56.9, ≥57.0 g/day.

**Table 2.** Characteristics of participants according to total MVPA among females

Characteristics	Total MVPA (MET-h/wk)^a^				

	Not meeting the guideline’s recommendation		Meeting the guideline’s recommendation		
	0	0.1–7.4	T1 (7.5–45.9)	T2 (46.0–125.9)	T3 (≥126.0)
Number of participants	4,190	1,294	13,180	12,347	13,990
Total MVPA, MET-h/week, median (IQR)	0	0.8 (0.8–3.4)	31.5 (11.3–42.0)	74.3 (63.0–94.5)	202.0 (152.6–269.3)
Non-leisure-time MVPA, MET-h/week, median (IQR)	0	0.0 (0.0–0.0)	10.5 (10.5–31.5)	63.0 (42.0–84.0)	184.6 (132.6–257.6)
Leisure-time MVPA, MET-h/week, median (IQR)	0	0.8 (0.8–3.4)	0.8 (0.0–4.0)	5.6 (0.8–17.6)	1.1 (0.0–13.6)
Age, years, mean (SD)	67.0 (7.9)	64.2 (8.0)	62.6 (8.1)	61.5 (7.4)	60.6 (6.9)
Smoking status, pack-years, *n* (%)					
Never smoker	3,615 (86.3)	1,175 (90.8)	12,113 (91.9)	11,433 (92.6)	12,917 (92.3)
Past smoker	63 (1.5)	30 (2.3)	247 (1.9)	220 (1.8)	158 (1.1)
0.1–30.0 pack-years	123 (2.9)	40 (3.1)	396 (3.0)	391 (3.2)	522 (3.7)
>30.0 pack-years	46 (1.1)	20 (1.6)	203 (1.5)	134 (1.1)	158 (1.1)
Missing	343 (8.2)	29 (2.2)	221 (1.7)	169 (1.4)	235 (1.7)
Alcohol consumption, g/week, *n* (%)					
Nondrinker	3,384 (80.8)	1,074 (83.0)	10,507 (79.7)	9,638 (78.1)	11,085 (79.2)
Occasional drinker	102 (2.4)	58 (4.5)	751 (5.7)	839 (6.8)	860 (6.2)
<150 g/week	149 (3.6)	69 (5.3)	959 (7.3)	998 (8.1)	1,053 (7.5)
150–299 g/week	43 (1.0)	26 (2.0)	298 (2.3)	277 (2.2)	287 (2.1)
300–449 g/week	32 (0.8)	16 (1.2)	128 (1.0)	132 (1.1)	162 (1.2)
≥450 g/week	66 (1.6)	16 (1.2)	294 (2.2)	288 (2.3)	319 (2.3)
Missing	414 (9.9)	35 (2.7)	243 (1.8)	175 (1.4)	224 (1.6)
Body mass index, kg/m^2^, *n* (%)					
<18.5	191 (4.6)	55 (4.3)	567 (4.3)	448 (3.6)	467 (3.3)
18.5–24.9	2,236 (53.4)	771 (59.6)	8,318 (63.1)	8,220 (66.6)	9,129 (65.3)
25.0–29.9	1,125 (26.9)	372 (28.8)	3,487 (26.5)	3,152 (25.5)	3,694 (26.4)
≥30.0	200 (4.8)	61 (4.7)	567 (4.3)	371 (3.0)	442 (3.2)
Missing	438 (10.5)	35 (2.7)	241 (1.8)	156 (1.3)	258 (1.8)
History of diabetes, *n* (%)					
No	3,892 (92.9)	1,214 (93.8)	12,445 (94.4)	11,825 (95.8)	13,449 (96.1)
Yes	298 (7.1)	80 (6.2)	735 (5.6)	522 (4.2)	541 (3.9)
Occupation, *n* (%)					
Agriculture/forestry/fishery	697 (16.6)	164 (12.7)	1,487 (11.3)	1,899 (15.4)	4,531 (32.4)
Hired/self-employed/professional/others	762 (18.2)	327 (25.3)	4,586 (34.8)	4,362 (35.3)	5,384 (38.5)
Housework	1,154 (27.5)	425 (32.8)	4,991 (37.9)	4,602 (37.3)	2,510 (17.9)
Non-employed/unemployed	797 (19.0)	247 (19.1)	1,447 (11.0)	881 (7.1)	520 (3.7)
Missing	780 (18.6)	131 (10.1)	669 (5.1)	603 (4.9)	1,045 (7.5)
Sitting time (hours/day), *n* (%)					
<1.0	641 (15.3)	401 (31.0)	3,519 (26.7)	2,590 (21.0)	3,931 (28.1)
1.0–2.9	317 (7.6)	120 (9.3)	3,451 (26.2)	4,107 (33.3)	4,766 (34.1)
3.0–4.9	251 (6.0)	115 (8.9)	2,349 (17.8)	2,839 (23.0)	2,395 (17.1)
5.0–6.9	145 (3.5)	76 (5.9)	1,475 (11.2)	1,278 (10.4)	1,032 (7.4)
≥7.0	224 (5.4)	97 (7.5)	1,415 (10.7)	969 (7.9)	1,059 (7.6)
Missing	2,612 (62.3)	485 (37.5)	971 (7.4)	564 (4.6)	807 (5.8)
Fruits and vegetable intake, g/day; median (IQR)^b^	369.4 (231.1–564.5)	395.0 (263.3–565.9)	409.8 (281.2–573.5)	433.8 (306.9–596.4)	425.1 (295.4–592.7)
Salted foods intake, g/day; median (IQR)^b^	9.0 (1.4–23.0)	10.9 (2.2–24.1)	10.3 (2.2–21.7)	10.8 (2.9–21.1)	11.0 (3.1–22.0)
Red and processed meat intake, g/day; median (IQR)^b^	26.1 (8.1–55.0)	28.9 (13.6–50.5)	29.8 (14.9–50.9)	29.5 (15.1–48.7)	29.2 (14.7–48.6)
Age at menopause, years, *n* (%)					
<45	457 (10.9)	165 (12.8)	1,358 (10.3)	1,230 (10.0)	1,488 (10.6)
45–49	1,081 (25.8)	365 (28.2)	3,585 (27.2)	3,330 (27.0)	3,958 (28.3)
50–54	1,522 (36.3)	511 (39.5)	5,778 (43.8)	5,671 (45.9)	6,109 (43.7)
≥55	256 (6.1)	71 (5.5)	681 (5.2)	598 (4.8)	644 (4.6)
Premenopausal	874 (20.9)	182 (14.1)	1,778 (13.5)	1,518 (12.3)	1,791 (12.8)
Use of hormone replacement therapy, *n* (%)					
No	3,031 (72.3)	1,101 (85.1)	11,744 (89.1)	11,227 (90.9)	12,589 (90.0)
Yes	78 (1.9)	34 (2.6)	294 (2.2)	261 (2.1)	336 (2.4)
Missing	1,081 (25.8)	159 (12.3)	1,142 (8.7)	859 (7.0)	1,065 (7.6)

IQR, interquartile range; MET-h/wk, metabolic equivalent hours per week; MVPA, moderate-vigorous physical activity; SD, standard deviation; T, tertile.

^a^Cut-off values of tertiles were calculated only among participants with a total MVPA of 7.5 MET-h/wk or higher.

^b^Food group intakes were energy-adjusted using the residual method and incorporated as categorical variables into regression models: fruits and vegetable <287.7, 287.7–418.8, 418.9–585.0, and ≥585.1 g/day; salted foods <2.5, 2.5–10.5, 10.6–21.7, and ≥21.8 g/day; red and processed meat <14.3, 14.3–29.1, 29.2–49.7, and ≥49.8 g/day.

### Follow-up and case identification

Participants were followed from the date of the response to the 10-year survey until the date of cancer diagnosis (11,344, 13.5%), death (12,633, 15.0%), moving out of a study area (3,818, 4.5%), loss to follow-up (35, 0.04%), or end of the follow-up period (67,568, 80.4%), whichever came first. The follow-up period ended on the following dates: December 31, 2012, in Osaka; December 31, 2013, in Kochi; December 31, 2014, in Nagasaki; and December 31, 2015, in the other areas. Changes in residence status and survival status were ascertained annually through the residential registry of each area. Incident cancer cases were mainly identified through patient records of major local hospitals and population-based cancer registries, with death certificates used in 535 individuals (Death Certificate Only [DCO] cases: 0.6%). Total cancer incidence, our primary outcome, was defined according to the International Classification of Diseases for Oncology, Third Edition (C00–C97). We also identified the following site-specific cancer incidences (major and minor sites)^22^: major cancer sites were gastric (C16), colorectal (C18–20), colon (C18), rectal (C19–20), lung (C34), breast (C50) for women, and prostate (C61) for men; and minor cancer sites were liver (C22), pancreatic (C25), kidney (C64), bladder (C67), endometrial (C54) for women, and ovarian (C56) for women.

### Statistical analysis

**Figure 1.**
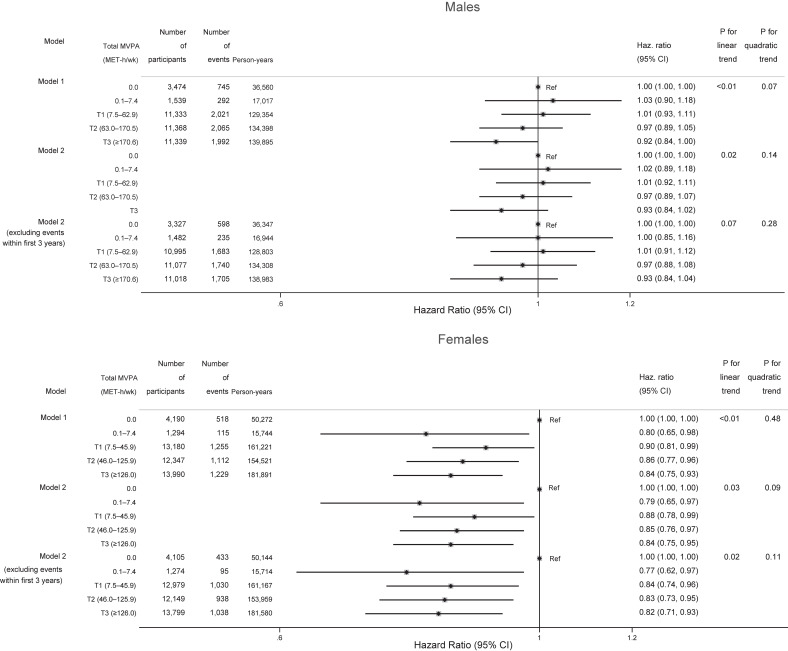
Associations between categorized amount of total MVPA and overall cancer by sex. Cut-off values of total MVPA were calculated in the same way as in Tables 1 or 2. Model 1 was adjusted for age and residential area and model 2 was adjusted for covariates in model 1 plus occupation, smoking status, alcohol consumption, BMI, diabetes, fruit and vegetable intake, sitting time, age at menopause (females only), and use of hormone replacement therapy (females only). Fruit and vegetable intake was categorized into quartiles and missing. Other covariates were treated in the same way as in Table 1 or 2. BMI, body mass index; CI, confidence interval; Haz. Ratio, hazard ratio; MET-hours/week, metabolic equivalent hours per week; MVPA, moderate-vigorous physical activity; T, tertile.

**Figure 2.**
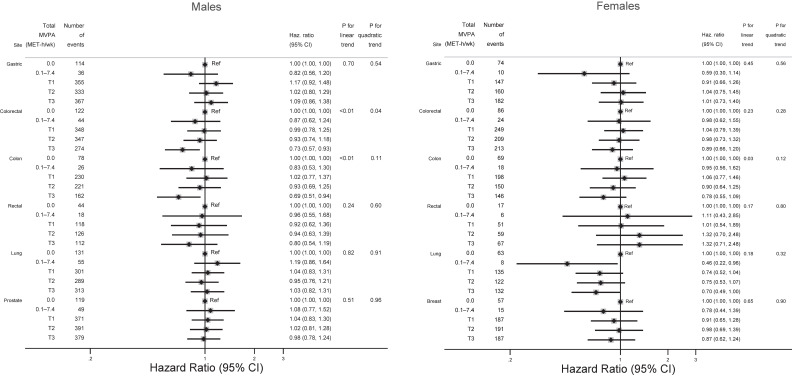
Associations between categorized amount of total MVPA and major site-specific cancers by sex. Cut-off values of total MVPA were calculated in the same way as in Table 1 or 2. The following covariates were adjusted for each site-specific cancer: age, residential area, occupation, smoking status, alcohol consumption, fruit and vegetable intake, sitting time, BMI, diabetes, and salted foods intake (gastric cancer); age, residential area, occupation, smoking status, alcohol consumption, fruit and vegetable intake, sitting time, BMI, diabetes, and red and processed meat intake intake (colorectal, colon, and rectal cancers); age, residential area, occupation, smoking status, alcohol consumption, fruit and vegetable intake, and sitting time (lung cancer), age, residential area, occupation, smoking status, alcohol consumption, fruit and vegetable intake, sitting time, BMI, and diabetes (prostate cancer); age, residential area, occupation, smoking status, alcohol consumption, fruit and vegetable intake, sitting time, BMI, diabetes, age at menopause, and use of hormone replacement therapy (breast cancer). BMI, body mass index; CI, confidence interval; Haz. Ratio, hazard ratio; MVPA, moderate-vigorous physical activity; T, tertile.

**Figure 3.**
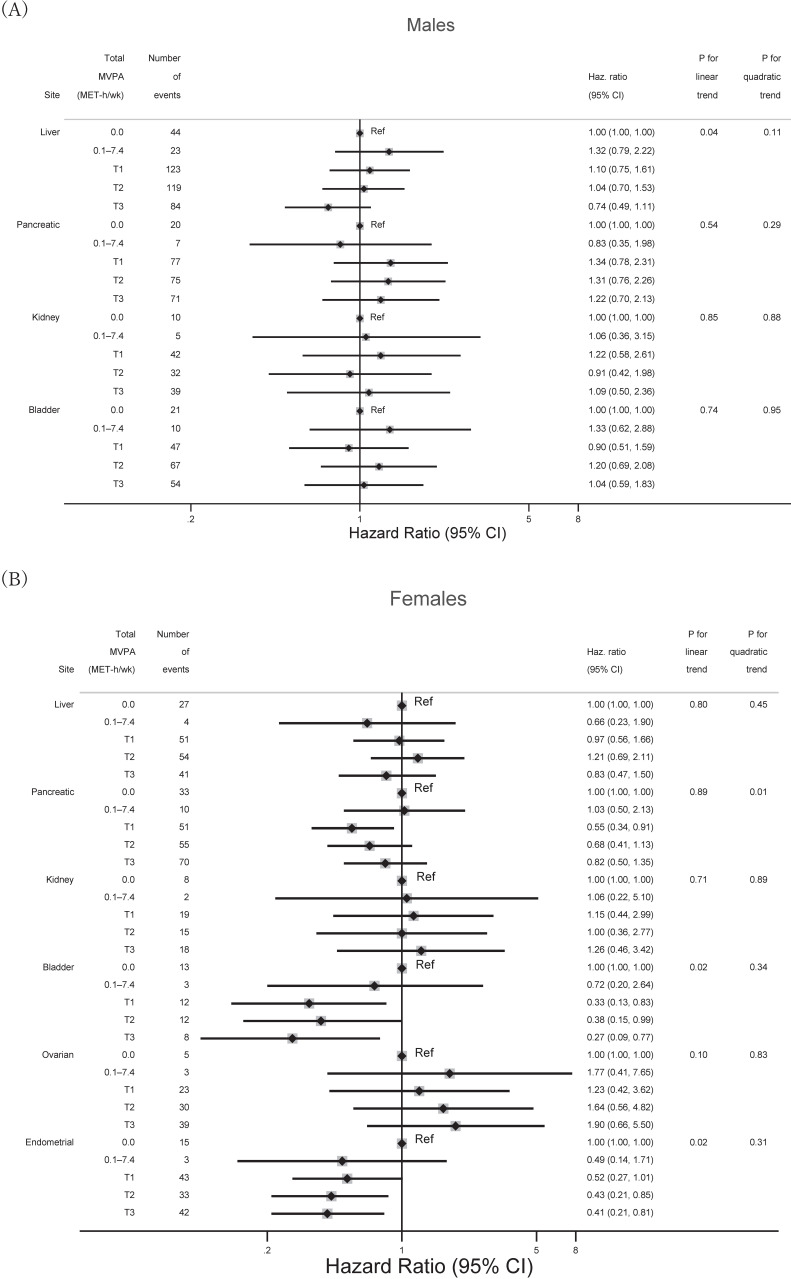
Associations between categorized amount of total MVPA and minor site-specific cancers. Cut-off values of total MVPA were the same as in Table 1 or 2. The following covariates were adjusted for each site-specific cancer: age, residential area, occupation, smoking status, alcohol consumption, fruit and vegetable intake, sitting time, BMI, and diabetes (liver, pancreatic, kidney, and bladder cancers); age, residential area, occupation, smoking status, alcohol consumption, fruit and vegetable intake, sitting time, BMI, diabetes, age at menopause, and use of hormone replacement therapy (ovarian and endometrial cancers). BMI, body mass index; CI, confidence interval; Haz. Ratio, hazard ratio; MVPA, moderate-vigorous physical activity; T, tertile.

All statistical analyses in the present study were conducted by sex because of differences between sexes in the biological mechanisms underlying cancer incidence and lifestyle, including physical activity, smoking, and drinking.^23^^,^^24^ The present study considered 7.5 MET-hours/week as the minimum recommended physical activity based on the WHO guidelines and a previous study.^4^ This recommended value (7.5) is the same for MPA and VPA because of the following calculations: 3 METs × 2.5 hours/week = 7.5 MET-hours/week for MPA or 6 METs × 1.25 hours/week = 7.5 MET-hours/week for VPA.^2^^,^^4^ Total MVPA was then divided into 0, 0.1–7.4 MET-hours/week, and tertiles (Ts) above 7.5 MET-hours/week. Associations of categorized total MVPA with overall and site-specific cancer incidences were examined using multivariable-adjusted Cox proportional hazard models, and hazard ratios (HRs) were estimated with their 95% confidence intervals (CIs). We selected 0 MET-hours/week as the reference because the WHO guidelines recommend any physical activity other than sedentary behavior.^2^ The present study treated death, moving out of a study area, and loss to follow-up as censored events. Two models (models 1 and 2) were built, and covariates were incorporated, as shown in the footnotes of Figure 1, Figure 2, and Figure 3. Tests for linear trends were performed by incorporating an ordinal variable scored as 0, 1, 2, 3, and 4 with increasing total MVPA as a single variable into models 1 and 2. To examine non-linear associations, tests for quadratic trends were also conducted by incorporating squared ordinal variables of total MVPA scored as 0, 1, 4, 9, and 16 as another single variable into the linear trend models. The likelihood ratio test calculated a *P* value for the product term between total MVPA and sex in model 2 to examine effect modification by sex. To examine the possibility of reverse causation, the present study estimated fully adjusted HRs of total cancer with categorized total MVPA, excluding events within the first 3 years of follow-up. Additionally, we examined the dose-response relationship between continuous total MVPA and total cancer using spline functions (eMaterial 2). We also conducted the following two additional analyses: 1) a stratified analysis by smoking or drinking habits (to address residual confounding from these habits) and industrial type (to address potential differences in the effects of non-leisure-time and leisure-time physical activity on cancer incidence); and 2) an analysis that examined associations of non-leisure-time and leisure-time MVPA (domain-specific MVPA) with total and major site-specific cancers. Due to the small number of participants with 0.1 to 7.5 MET-hours/week of non-leisure-time MVPA and the small number of cases with 0.1 to 7.5 MET-hours/week of leisure-time MVPA, non-leisure-time MVPA was categorized into less than 7.5 MET-hours/week and tertiles above 7.5 MET-hours/week, and leisure-time MVPA was categorized into 0 MET-hours/week and tertiles above 0 MET-hours/week based on the distributions of these MVPAs.

Finally, regarding the associations of categorized amount of total MVPA with overall and site-specific cancers, we conducted four sensitivity analysis. Details of these sensitivity analyses are described in eMaterial 3. All statistical tests were two-sided and considered a *P* value <0.05 as indicating statistical significance. All statistical analyses were done using SAS software version 9.4 (SAS Institute, Cary, NC, USA) and forest plots were created using Stata version 17.0 (StataCorp, College Station, TX, USA).

## RESULTS

Median for total reported MVPA for the 39,053 males and 45,001 females were 84.0 (interquartile range [IQR], 31.5–196.0) and 63.8 (IQR, 22.1–143.9) MET-hours/week, respectively. During a median follow-up of 13.0 years, 7,115 (18.2%) and 4,229 (9.4%) total cancer events occurred among males and females, respectively. Table 1 and Table 2 shows participant characteristics according to total MVPA by sex. Participants of both sexes with a higher total MVPA were likely to be younger; have a normal body mass index; have no history of diabetes; to be working; spend less time sitting; and consume higher amounts of fruits and vegetables, salted foods and meat. Additionally, active males tended to consume higher amounts of cigarettes and alcohol, whereas active females tended to have experienced menopause at a younger age.

Figure 1 shows associations between the categorized amount of total MVPA and overall cancer by sex. Among males, an inverse dose-response relationship was found between total MVPA and overall cancer (*P* for linear trend = 0.02), but the risk reduction was non-significant even in the highest MVPA category compared with no total MVPA. Among females, in contrast, the risk of total cancer was reduced even when they had engaged in lower total MVPA than the recommended amount compared with a total MVPA of 0 MET-hours/week: HRs were 0.79 (95% CI, 0.65–0.97), 0.88 (95% CI, 0.78–0.99), 0.85 (95% CI, 0.76–0.97), and 0.84 (95% CI, 0.75–0.95) for 0.1–7.4 MET-hours/week, T1 (7.5–45.9 MET-hours/week), T2 (46.0–125.9), and T3 (≥126.0), respectively (*P* for linear trend = 0.03). Although *P* for linear trend was significant among females, HRs were stable for total MVPA over 0.1–7.4 MET-hours/week. *P* for quadratic trends were non-significant in both sexes. The *P* value for the product term by total MVPA and sex was 0.29, and no effect modification by sex was found. Analysis excluding events within the first 3 years of follow-up yielded almost the same results as the main analysis. Spline curves showed an inverse association between total MVPA and overall cancer among both sexes (eFigure 3). The curve for females confirmed an L-shaped dose-response relationship shown by the categorized analysis (Figure 1), but *P* values for non-linearity were not significant among both sexes.

Figure 2 shows associations between the categorized amount of total MVPA and major site-specific cancers among each sex. Whereas engaging in higher total MVPA was inversely associated with reduced risk of colon cancer among males, a marginal inverse association was found with lung cancer among females. Figure 3 shows associations between total MVPA and the minor cancer sites. In these analyses, engaging in higher total MVPA was inversely associated with risk of bladder and endometrial cancers among females.

**Figure 4.**
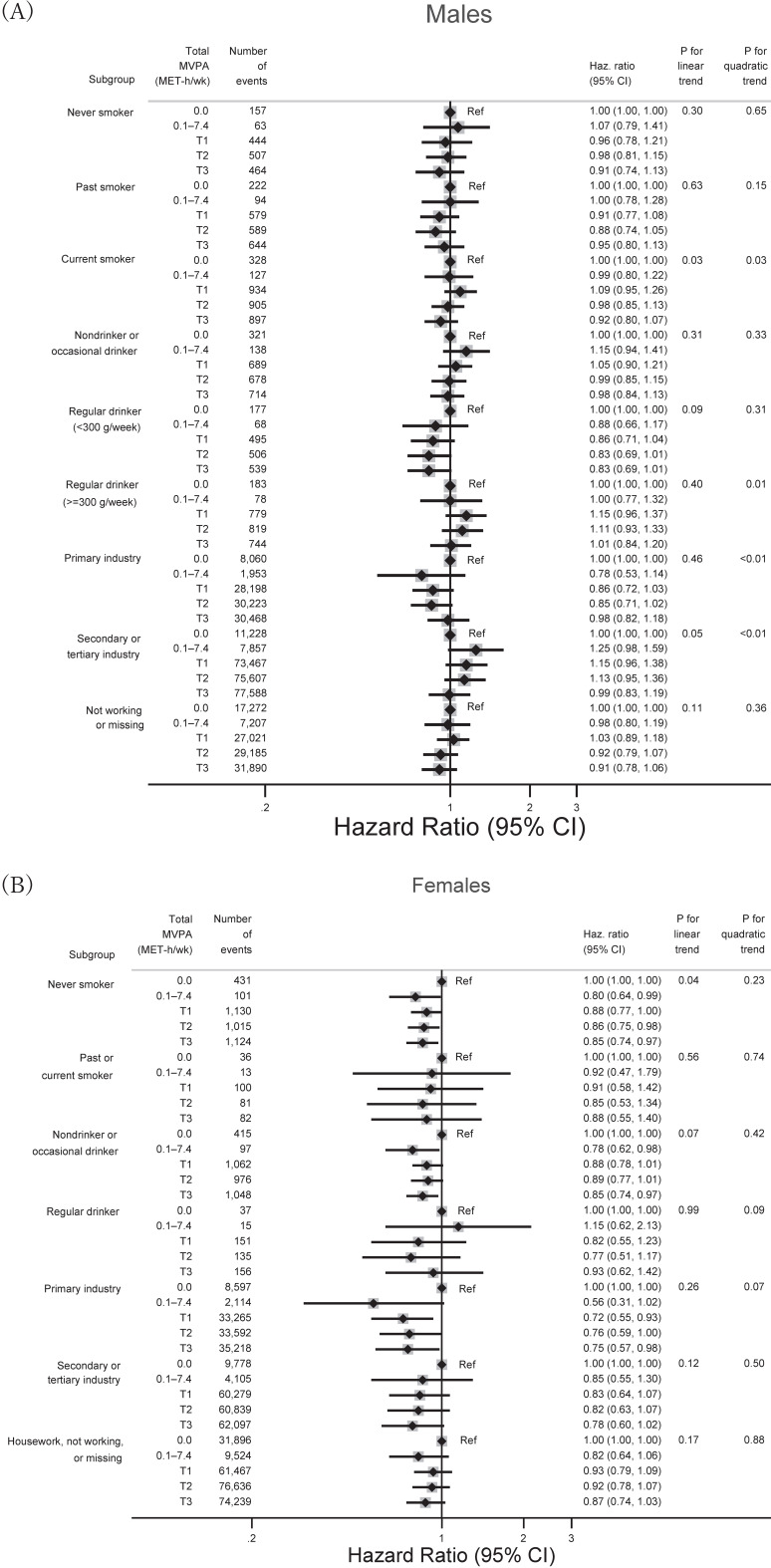
Associations between categorized amount of total MVPA and overall cancer by smoking status, alcohol consumption, and industrial type. Each industry type consisted of the following occupations: agriculture, fishery, and forestry for primary industry; hired, self-employed, professional, and others for secondary or tertiary industry. Cut-off values of total MVPA were calculated in the same way as in Table 1. Models were adjusted for the same covariates as those in model 2 of Figure 1 (total cancer) or the model of Figure 2 (site-specific cancers). CI, confidence interval; Haz. Ratio, hazard ratio; MVPA, moderate-vigorous physical activity; T, tertile.

**Figure 5.**
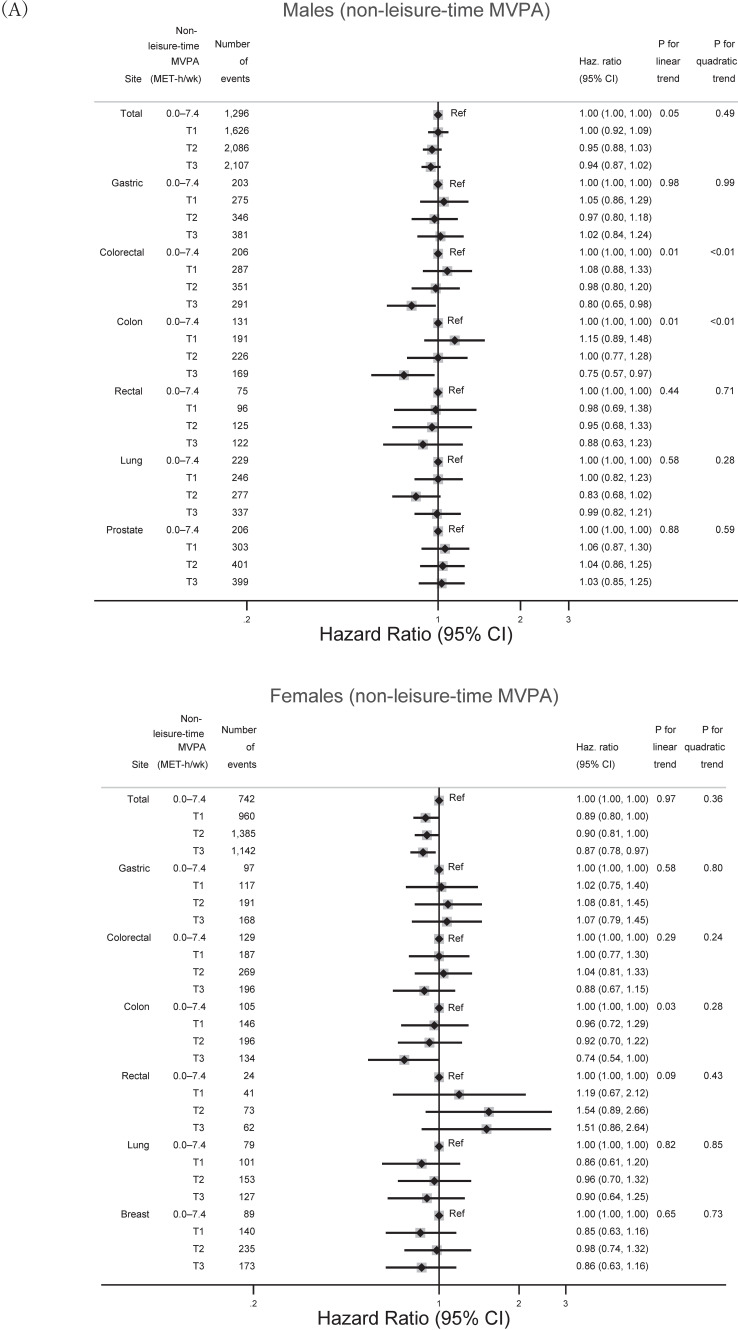

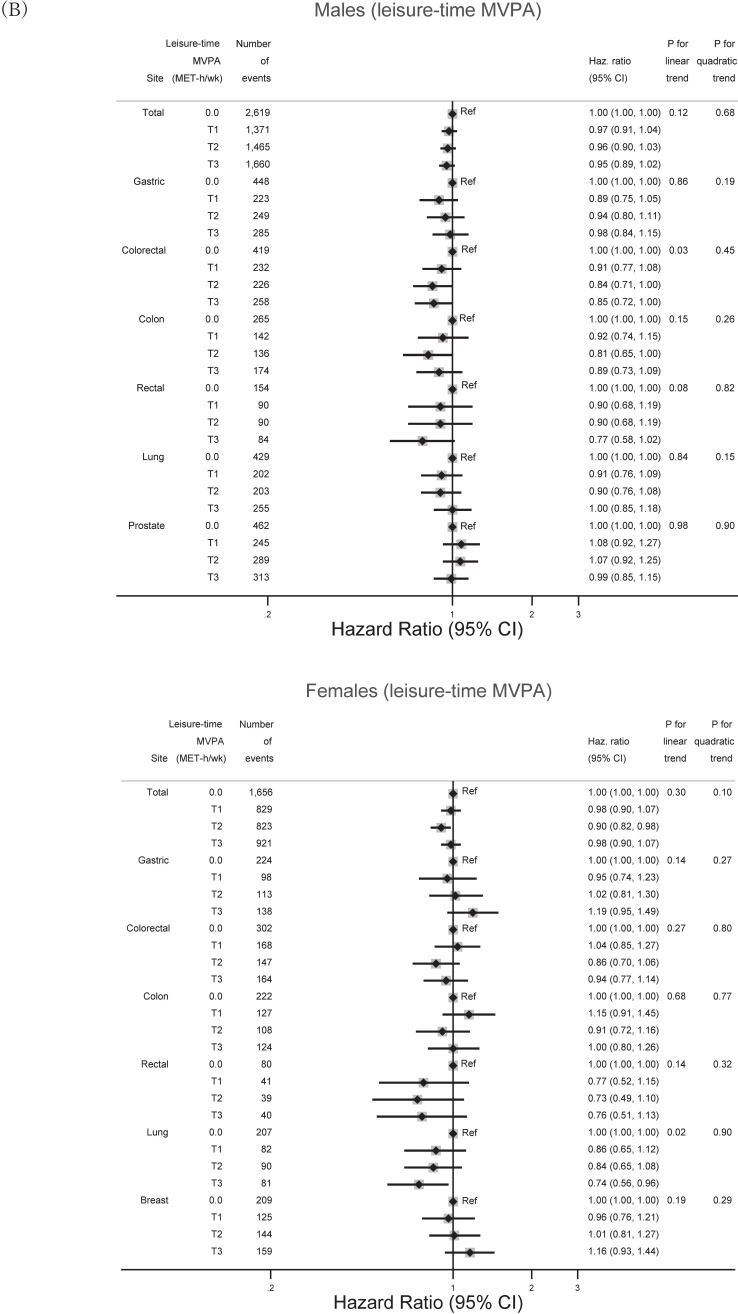
Associations between categorized amount of domain-specific MVPA and total and major site-specific cancers by sex. Non-leisure-time MVPA was categorized into less than 7.5 MET-hours/week and tertiles above 7.5 MET-hours/week, and leisure-time MVPA was categorized into 0 MET-hours/week, and tertiles above 0 MET-hours/week based on the distributions of these MVPAs. Models were adjusted for the same covariates as those in model 2 of Figure 1 (total cancer) or the model of Figure 2 (site-specific cancers). CI, confidence interval; Haz. Ratio, hazard ratio; MVPA, moderate-vigorous physical activity; T, tertile.

Figure 4 shows associations between the categorized amount of total MVPA and overall cancer by smoking status, alcohol consumption, and industrial type. Whereas among females, total MVPA generally showed inverse associations with overall cancer, point estimates were above unity in male regular drinkers (300 g/week) and male secondary or tertiary industrial workers. Figure 5 shows associations between categorized amount of domain-specific MVPA and total and major site-specific cancers by sex. The directions of the associations were generally the same as those with total MVPAs (Figure 1 and Figure 2).

Results in the other sensitivity analyses using quintile categories of total MVPA, excluding non-leisure-time walking, with multiple imputation analysis and adjustment for other covariates were in a similar direction to the main results (eFigure 4, eFigure 5, eFigure 6, and eFigure 7).

## DISCUSSION

This prospective cohort study with a follow-up of 13 years investigated associations of overall and site-specific cancers with total MVPA from any domain, which have been understudied. Among males, the inverse association for total cancer was non-significant, even at the highest category of total MVPA. Among females, in contrast, total MVPA was associated with reduced risk even at a lower level than the recommended amount, and no further risk reduction was observed with increasing MVPA. Moreover, higher levels of total MVPA were associated with reduced risks of colon cancer among males and bladder and endometrial cancers among females.

To date, total MVPA has been associated with lower risks of site-specific cancer incidences,^25^^–^^28^ but relations between total MVPA and overall cancer incidence are uncertain.^1^ Nevertheless, a pooled analysis of nine cohort studies^4^ reported that a leisure-time MVPA of 7.5–15.0 MET-hours/week (amount recommended by WHO) was associated with significantly reduced risk of 7 of the 15 site-specific cancers examined, which partly agrees with our present results for total cancer among females. Regarding dose-response relationships, this pooled analysis^4^ demonstrated that the dose-response relationships between leisure-time MVPA and several site-specific cancer incidences were approximately linear. In the present study, although the spline curve for females looked non-linear, *P* for quadratic trend and *P*-nonlinearity were not significant between total MVPA and overall cancer in both sexes, which is partly consistent with the results of the pooled analysis.^4^

Little is known about the sex-specific effects of total MVPA on overall cancer. In our present study, although the product term between total MVPA and sex was not significant, the inverse association between total MVPA and overall cancer was more pronounced among females than males. In contrast, a meta-analysis of cohort studies reported that the protective effect of leisure-time physical activity on total cancer was almost the same in magnitude between sexes.^3^ We speculated that males’ higher prevalence of unhealthy lifestyles, including smoking and drinking, could have negated the beneficial effect of physical activity. In fact, in additional stratified analysis by smoking and drinking status, point estimates among male regular drinkers were above unity (Figure 4). Additionally, this difference between sexes may involve differential exposure to other environmental (eg, infection, hazardous occupational exposure) or biological factors (eg, sex hormones).^23^^,^^24^ Males are more likely to engage in physically strenuous occupations than females, and these tasks are suggested to have neutral (eg, insufficient intensity to enhance aerobic capacity) or harmful effects (eg, insufficient recovery time leading to chronic inflammation and lack of control by workers) on health.^29^ Nevertheless, an umbrella review of systematic reviews found no harmful effects of occupational physical activity on site-specific cancers.^7^ On the other hand, our additional analysis with stratification by industrial type showed a marginally positive association between total MVPA and overall cancer in male secondary or tertiary industry workers, which may have diluted a beneficial effect of total MVPA (Figure 4). Among females, we found significant or marginal associations of total MVPA with bladder, endometrial, colon, and lung cancers, which may have combined to lower the risk of total cancer.

In the present study, total MVPA was inversely associated with risk of colon cancer among males and bladder and endometrial cancers among females. Evidence summaries differ in their evaluation of the strength of evidence across cancer sites; however, they consistently conclude that the inverse associations between physical activity (not limited to MVPA) and risks of colorectal and breast cancers are supported by strong evidence.^1^^,^^30^^,^^31^ Moreover, meta-analyses have demonstrated inverse associations of physical activity (including moderate and vigorous intensity physical activity) with bladder^19^ and endometrial cancers.^32^ Our results generally confirmed the findings of these evidence summaries and meta-analyses in a Japanese community-dwelling population. In contrast, the present study did not find a negative association between total MVPA and breast cancer. Although a meta-analysis reported a significant inverse association between total physical activity and breast cancer among postmenopausal females only,^33^ an additional analysis in our study showed a preventive effect of total MVPA on premenopausal breast cancer (*N* = 6,143; HRs of total MVPA of 0.34; 95% CI, 0.08–1.55, 0.51; 95% CI, 0.24–1.08, 0.44; 95% CI, 0.20–0.96, and 0.37; 95% CI, 0.17–0.82 for 0.1–7.4 MET-hours/week, T1, T2, and T3, respectively), but no effect on postmenopausal breast cancer (*N* = 38,858; HRs of total MVPA of 0.92; 95% CI, 0.49–1.74, 1.01; 95% CI, 0.68–1.49, 1.17; 95% CI, 0.79–1.73, and 1.03; 95% CI, 0.70–1.54 for 0.1–7.4 MET-hours/week, T1, T2, and T3, respectively). Ascertaining the factors responsible for these differences in breast cancer incidence between our present and these previous studies may require stratified analysis by other variables, including hormone receptors. Regarding colorectal cancer, the association with total MVPA was not significant among females. A systematic review of observational studies conducted in Japanese populations^34^ suggested that the protective effect of physical activity on colorectal cancer risk is generally more consistent and pronounced in males than in females. This sex difference has also been observed in other populations.^34^ One possible explanation for the sex difference is that physical activity among females, especially in the Japanese context, often consists of household activities, which are less salient and thus more difficult to recall accurately in self-reported questionnaires.^34^

Physical activity may prevent cancer development and progression through several biological mechanisms: 1) reducing body fat, 2) improving insulin sensitivity and fasting insulin levels, 3) promoting secretion of antimitotic and apoptotic adipokines, such as adiponectin, with reducing antiapoptotic adipokines, such as leptin, 4) ameliorating chronic inflammation, 5) decreasing levels of endogenous estrogens and androgens, 6) reducing oxidative stress and increasing antioxidant enzymes, and 7) enhancing immune function.^35^^–^^37^ Of note, few specific mechanisms have been identified for aerobic or resistance exercise to date.

The strengths of the present study include the examination of total MVPA as exposure, prospective follow-up over 13 years with a sizable Japanese community-dwelling population, accurate identification of cancer cases, extensive adjustment of potential confounding factors, and examination of multiple site-specific cancers as outcomes. Our inclusion of non-leisure-time physical activity and confirmation of the inverse association among females is likely of particular value in reviewing the current evidence given that non-leisure-time physical activity accounts for a large percentage of daily activities for most individuals. Moreover, the above-mentioned pooled analysis did not include the Japanese population,^4^ and common site-specific cancers differ between Japan and Western countries (eg, gastric cancer is more common in Japan than in Western countries).^11^ Therefore, the present study provides the evidence applicable to the Japanese population. However, the following limitations should also be considered when interpreting the findings. First, although our physical activity questionnaire was reported to have acceptable reliability and validity, it tended to overestimate MVPA compared with measurement using activity records.^9^ Therefore, the actual dose-response relationships between MVPA and total cancer can be located more to the left (at lower MVPA) than those provided by the present study. Second, the present study evaluated physical activity only at the start of follow-up (10-year follow-up survey). Some participants likely changed their physical activity patterns, possibly leading to misclassification of physical activity. Although our 5-year follow-up survey also measured physical activity, this measurement included only one MVPA item with only three categories, which prevented accurate MVPA evaluation. Accordingly, we did not use the information from the 5-year follow-up survey. Third, reverse causation may have affected the estimates, wherein individuals with non-symptomatic cancer were less likely to engage in physical activity. However, results excluding events within the first 3 years of the follow-up were materially unchanged. Fourth, the low number of events and the multiple testing prevented the drawing of definitive conclusions for site-specific cancers. Fifth, missing values for several variables may have biased estimates, although the results of the additional analysis using multiple imputation showed the same direction as those of the main analysis. Sixth, the present study adjusted for covariates that were measured at the 10-year follow-up survey, which may have led to adjusting for mediators between total MVPA and cancer incidence. Seventh, the present study did not have information on some potential confounding factors in all participants (eg, socioeconomic status [SES] other than occupation and *Helicobacter pylori* and hepatitis virus infection), which could have caused residual confounding. Regarding SES, however, lifestyle factors such as smoking, drinking, and dietary habits may exist in the middle of the path from SES to physical activity, and adjusting for these factors would partly control confounding from SES. Eighth, the present study participants consisted of Japanese community-dwelling adults with a median age of 61.4 years (IQR, 55.4–67.5) who answered a questionnaire and participated in the 10-year follow-up survey. Thus, our results may not be generalizable to populations with different ages, genetic backgrounds, or lifestyles.

In conclusion, total MVPA was associated with a reduced risk of overall cancer incidence in females, but not in males. This association also varied by cancer site. Nevertheless, even a small amount of MVPA, regardless of domain, may contribute to cancer prevention in women. To promote physical activity at the population level, individual behavioral change should be supported by environmental- and policy-level interventions, such as infrastructure for active transportation (eg, walking and bicycling). Future studies incorporating objective measures like accelerometry are needed to elucidate the relationship between physical activity and total cancer risk.

## SUPPLEMENTARY MATERIAL

eMaterial 1. Physical activity questionnaire used in the Japan Public Health Center-based Prospective Study

eMaterial 2. Methods of dose-response analysis

eMaterial 3. Methods and results of sensitivity analyses

eFigure 1. Flowchart showing the selection of eligible participants. JPHC study, Japan Public Health Center-based Prospective Study.

eFigure 2. Directed acyclic graphs showing relationships among total MVPA, site-specific cancers, and potential confounding factors. SES was unmeasured. FV, fruits and vegetables; MVPA, moderate-to-vigorous physical activity; SES, socioeconomic status.

eFigure 3. Spline functions showing the relationship between continuous MVPA and total cancer incidence by sex. Models were adjusted for the same covariates as those in model 2 of Table 2.

eFigure 4. Associations between quintiles of total MVPA and overall and major site-specific cancers.

eFigure 5. Associations between categorized amount of MVPA excluding non-leisure-time walking and total and major site-specific cancers.

eFigure 6. Associations of categorized amount of total MVPA with overall and major site-specific cancers in each sex, with multiple imputation of missing values.

eFigure 7. Associations between categorized amount of MVPA and total and major site-specific cancers with additional covariate adjustment.
