# Supplementary material for: Moderate-to-vigorous Physical Activity and Total and Site-specific Cancer: The Japan Public Health Center-based Prospective Study
Source: J Epidemiol. 2026 Mar 5;36(3):94–106. doi: 10.2188/jea.JE20250041 (PMC12893842; doi:10.2188/jea.JE20250041)
Supplement: Supplementary file 1 [file je-36-094-s001.pdf]

eMaterial 1. Physical activity questionnaire used in the Japan Public Health Center-based Prospective Study

1. Non-leisure-time physical activity

Please report how much time you spent doing the tasks below during working time on a typical day in the previous year. Working time includes time spent commuting and housekeeping.

Task	None	<1 hour	1.0–2.9 hours	3.0–4.9 hours	5.0–6.9 hours	7.0–8.9 hours	9.0–10.9 hours	≥11.0 hours
Sitting	□	□	□	□	□	□	□	□
Standing	□	□	□	□	□	□	□	□
Walking	□	□	□	□	□	□	□	□
Vigorous tasks	□	□	□	□	□	□	□	□

2. Leisure-time physical activity

This section asks about your physical activity during leisure periods. Please report the frequency and duration of each of the activities below per session during the previous year.

Task	Frequency				
	<1 time/month	1–3 times/month	1–2 times/week	3–4 times/week	Almost daily
Strolling	□	□	□	□	□
Brisk walking	□	□	□	□	□
Moderate intensity tasks such as playing golf and gardening	□	□	□	□	□
Vigorous intensity tasks such as jogging, aerobics, and swimming	□	□	□	□	□

Task	Duration per session					
	<30 minutes	30–59 minutes	1.0–1.9 hours	2.0–2.9 hours	3.0–3.9 hours	≥4.0 hours
**Strolling**	☐	☐	☐	☐	☐	☐
**Brisk walking**	☐	☐	☐	☐	☐	☐
**Moderate intensity tasks such as playing golf and gardening**	☐	☐	☐	☐	☐	☐
**Vigorous intensity tasks such as jogging, aerobics, and swimming**	☐	☐	☐	☐	☐	☐

eMaterial 2. Methods of dose-response analysis

The present study examined dose-response relationships between continuous total moderate-to-vigorous physical activity (MVPA) and overall cancer incidence using fully adjusted Cox proportional hazard models with restricted cubic splines that included the same covariates in model 2 of **Figure 1**. These spline functions used 3−6 knots, with the optimal numbers of knots determined based on Akaike's Information Criterion. We selected the positions of knots based on the World Health Organization (WHO)'s recommendation (total MVPA of 7.5 metabolic equivalent of task [MET]-hours/week) and cutoff values of the main analysis using categorized total MVPA. Knots based on the WHO's recommendation were placed as follows: three knots, 3.75, 7.5, and 300 MET-hours/week; four knots: 3.75, 7.5, 150, and 300 MET-hours/week; five knots, 3.75, 7.5, 75, 150, and 300 MET-hours/week; and six knots, 3.75, 7.5, 75, 150, 225, and 300 MET-hours/week. Knots based on cutoff values of the main analysis were placed as follows: males, 3.75, 7.5, 63.0, and 170.6 MET-hours/week; and females, 3.75, 7.5, 46.0, and 126.0 MET-hours/week. We included a knot of 3.75 MET-hours/week because a previous pooled analysis reported that risks of some site-specific cancers reduced until 7.5−15.0 MET-hours/week, after which no further risk reduction was observed.[1] Tests for nonlinearity were performed using the likelihood ratio test by comparing models with and

without spline terms. The present study considered analyses using the categorized total MVPA as primary because physical activity measured using our questionnaire is appropriate for treatment as an ordinal scale rather than a ratio scale due to its probable overestimation.

REFERENCES

1. Matthews CE, Moore SC, Arem H, Cook MB, Trabert B, Håkansson N, et al. Amount and Intensity of Leisure-Time Physical Activity and Lower Cancer Risk. J Clin Oncol. 2020;38(7):686-97, 10.1200/JCO.19.02407.

eMaterial 3. Methods and results of sensitivity analyses

Regarding associations of the categorized amount of total moderate-to-vigorous physical activity (MVPA) with overall and site-specific cancers, we conducted the following four sensitivity analyses: sensitivity analysis 1) categorized total MVPA into quintiles to investigate the influence using relatively small number of reference categories (0 MET-hours/week) (eFigure 4); sensitivity analysis 2) excluded non-leisure-time walking from total MVPA calculation because our questionnaire did not specify speed for non-leisure-time walking (eFigure 5); sensitivity analysis 3) incorporated multiple imputation which included the 747 males and 803 females (150 and 92 cases of total cancer) excluded from the main analyses due to missing values in physical activity (eFigure 6); and sensitivity analysis 4) additionally incorporated use of antihypertensive and cholesterol-lowering drugs, parental history of cancer, age of menarche, and parity as covariates (age of menarche and parity were adjusted only in breast cancer) (eFigure 7). Sensitivity analysis 4) relied on self-reported information from the baseline survey of the Japan Public Health Center-based Prospective Study on parental history of cancer, age of menarche, and parity (measured 10 years before the start of the present follow-up of cancer incidence). In contrast, the use of antihypertensive and cholesterol-lowering drugs was based on self-reported information from the 10-year survey.

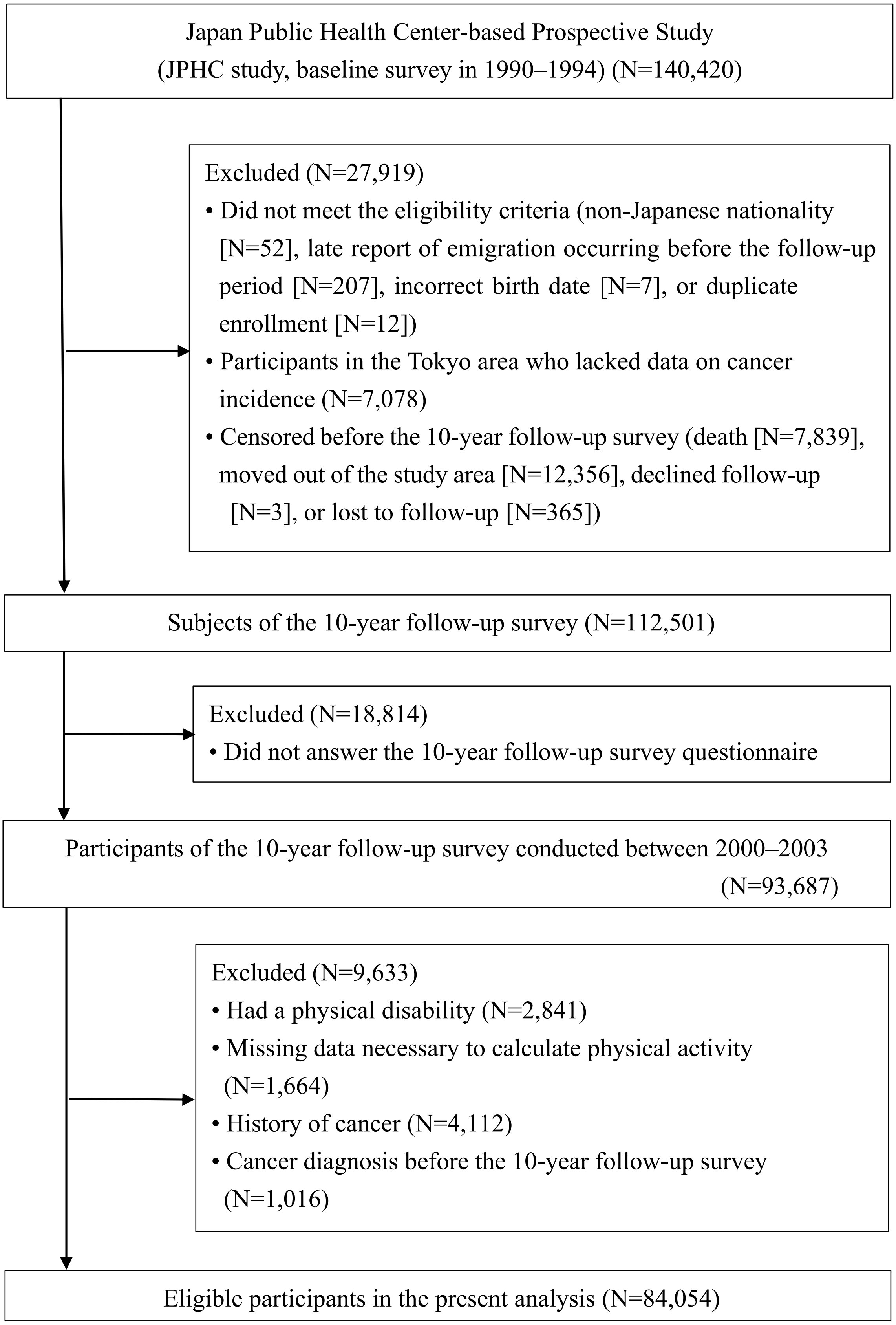

eFigure 1. Flowchart showing the selection of eligible participants. JPHC study, Japan Public Health Center-based Prospective Study.

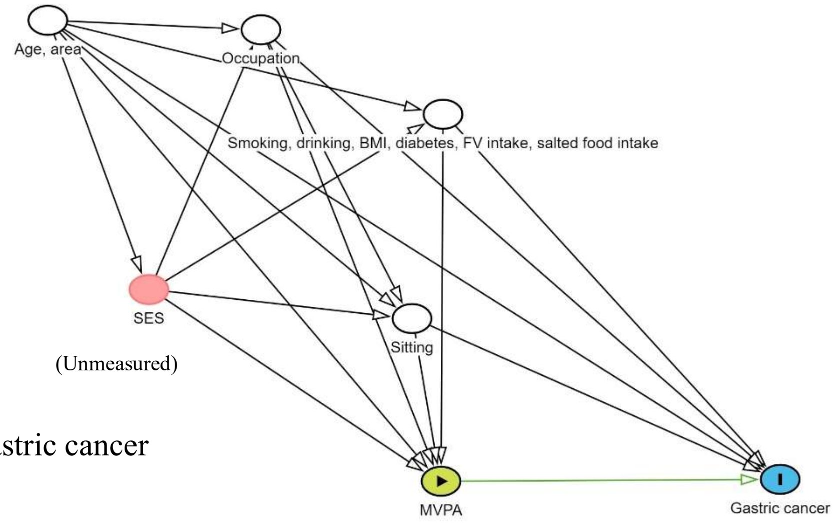

A. Gastric cancer

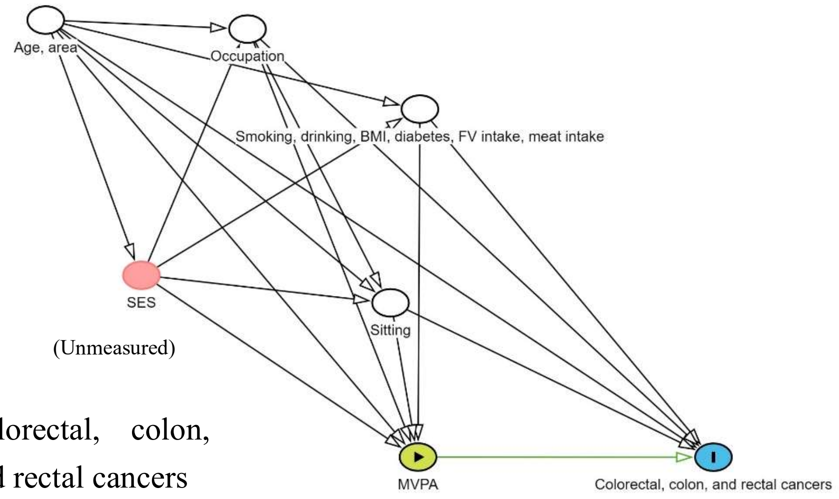

B. Colorectal, colon, and rectal cancers

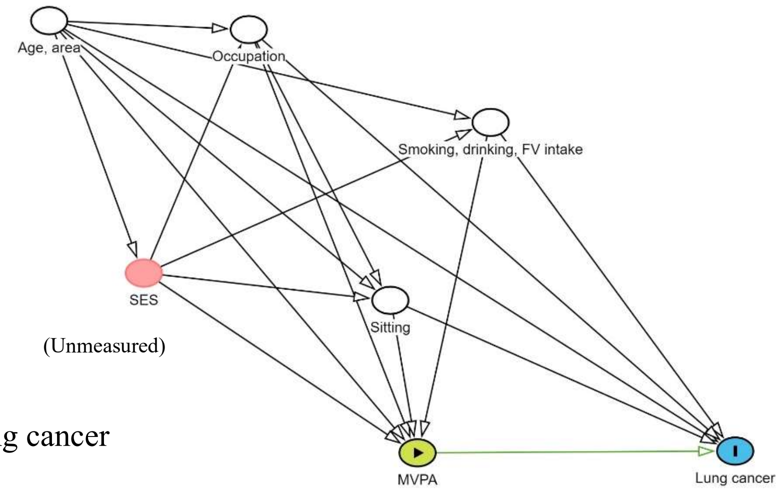

C. Lung cancer

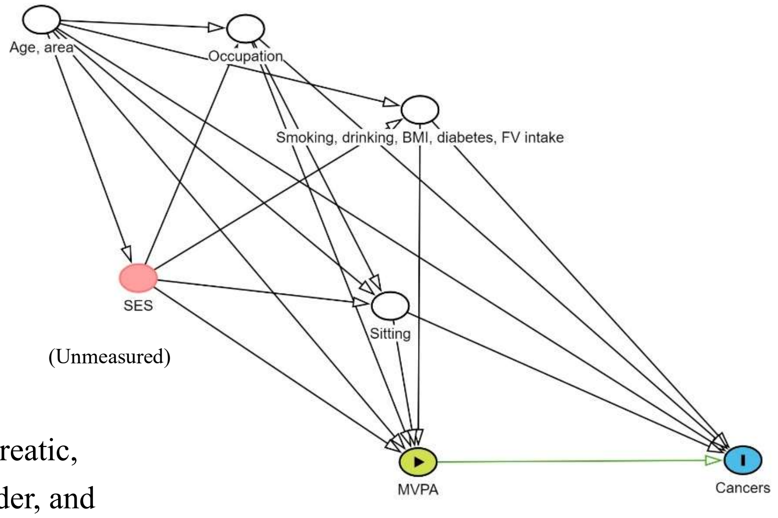

D. Liver, pancreatic, kidney, bladder, and prostate cancers

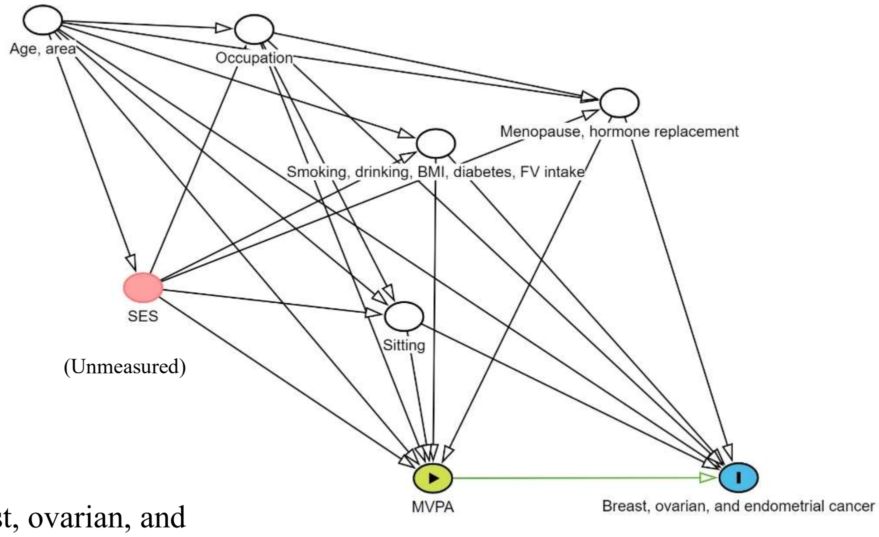

E. Breast, ovarian, and endometrial cancers

eFigure 2. Directed acyclic graphs showing relationships among total MVPA, site-specific cancers, and potential confounding factors. SES was unmeasured. FV, fruits and vegetables; MVPA, moderate-to-vigorous physical activity; SES, socioeconomic status.

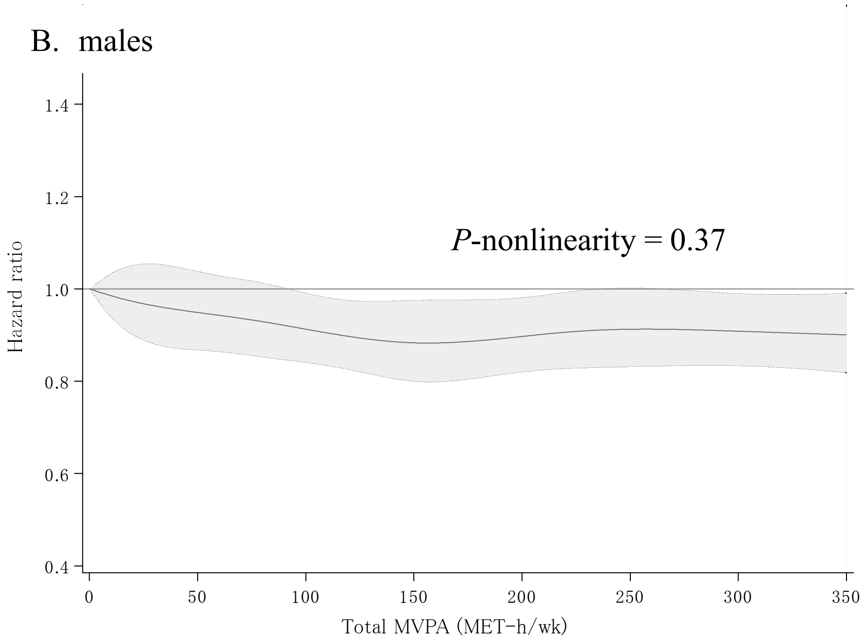

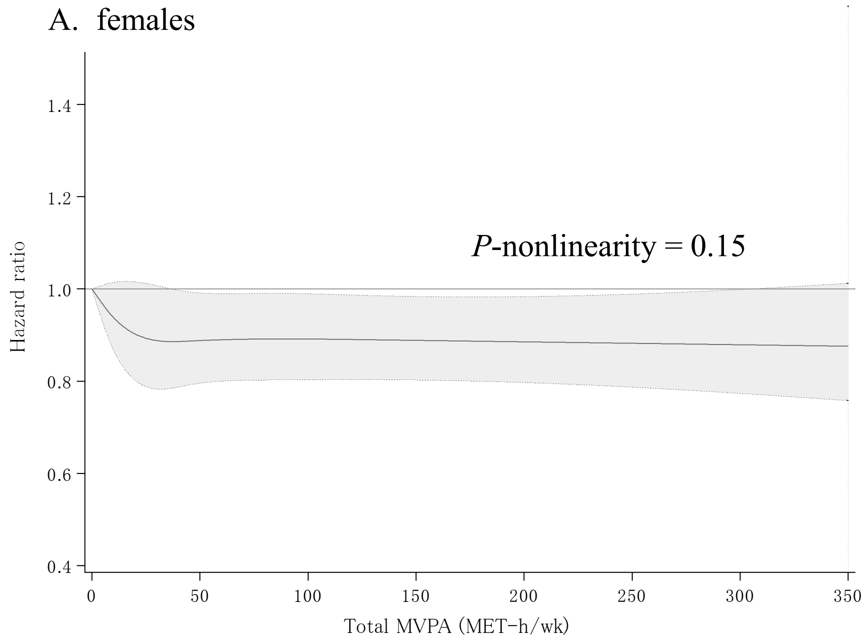

eFigure 3. Spline functions showing the relationship between continuous MVPA and total cancer incidence by sex. Models were adjusted for the same covariates as those in model 2 of Table 2.

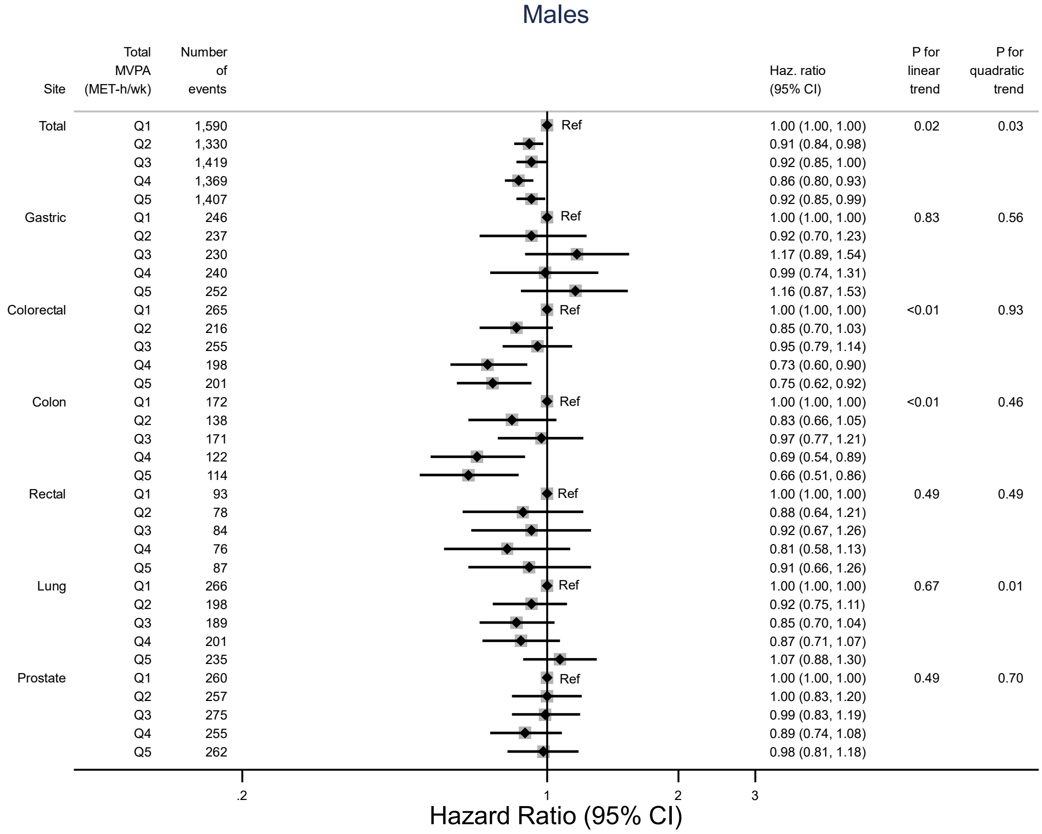

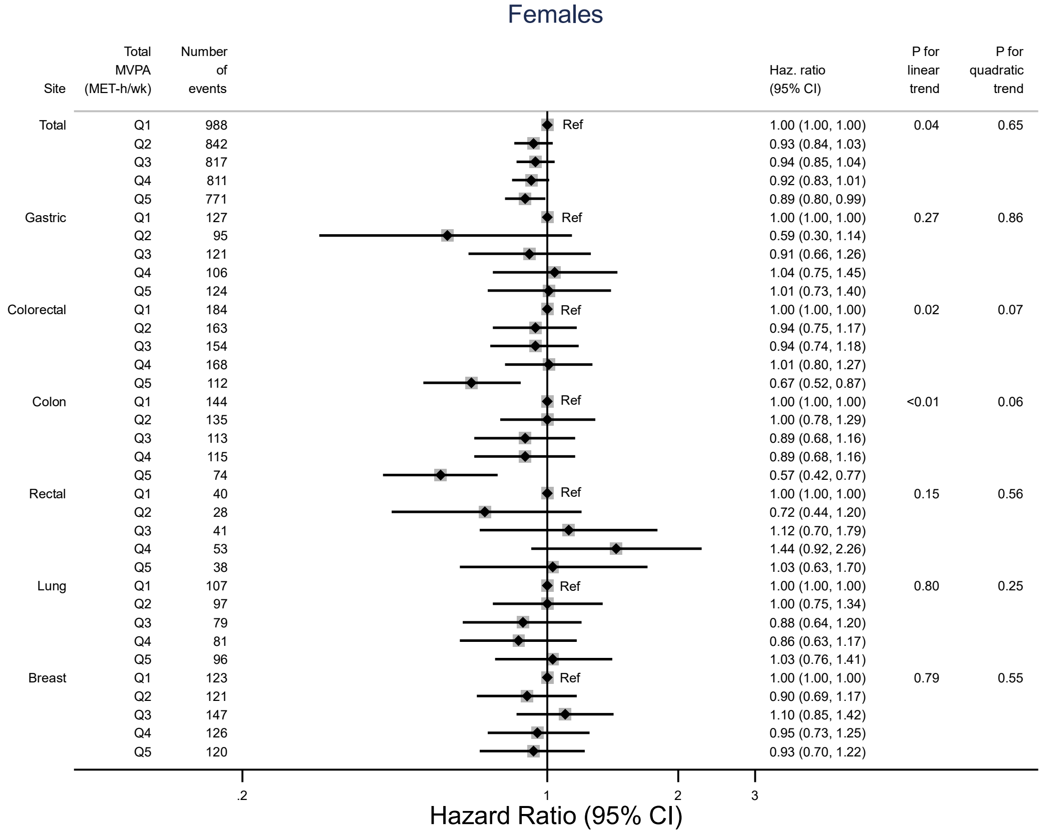

eFigure 4. Associations between quintiles of total MVPA and overall and major site-specific cancers.

Ranges of total MVPA quintiles were as follows: 0.0–14.9, 15.0–52.3, 52.4–123.7, 123.8–233.7, and ≥233.8 MET-h/wk among males; 0.0–11.5, 11.6–43.4, 43.5–86.7, 86.8–169.2, and ≥169.3 MET-h/wk among females. Models were adjusted for the same covariates as those in model 2 of Figure 1 (total cancer) or the model of Figure 2 (site-specific cancers). CI, confidence interval; Haz. Ratio, hazard ratio; MET-h/wk, metabolic equivalent hours per week; MVPA, moderate-vigorous physical activity; Q, quintile.

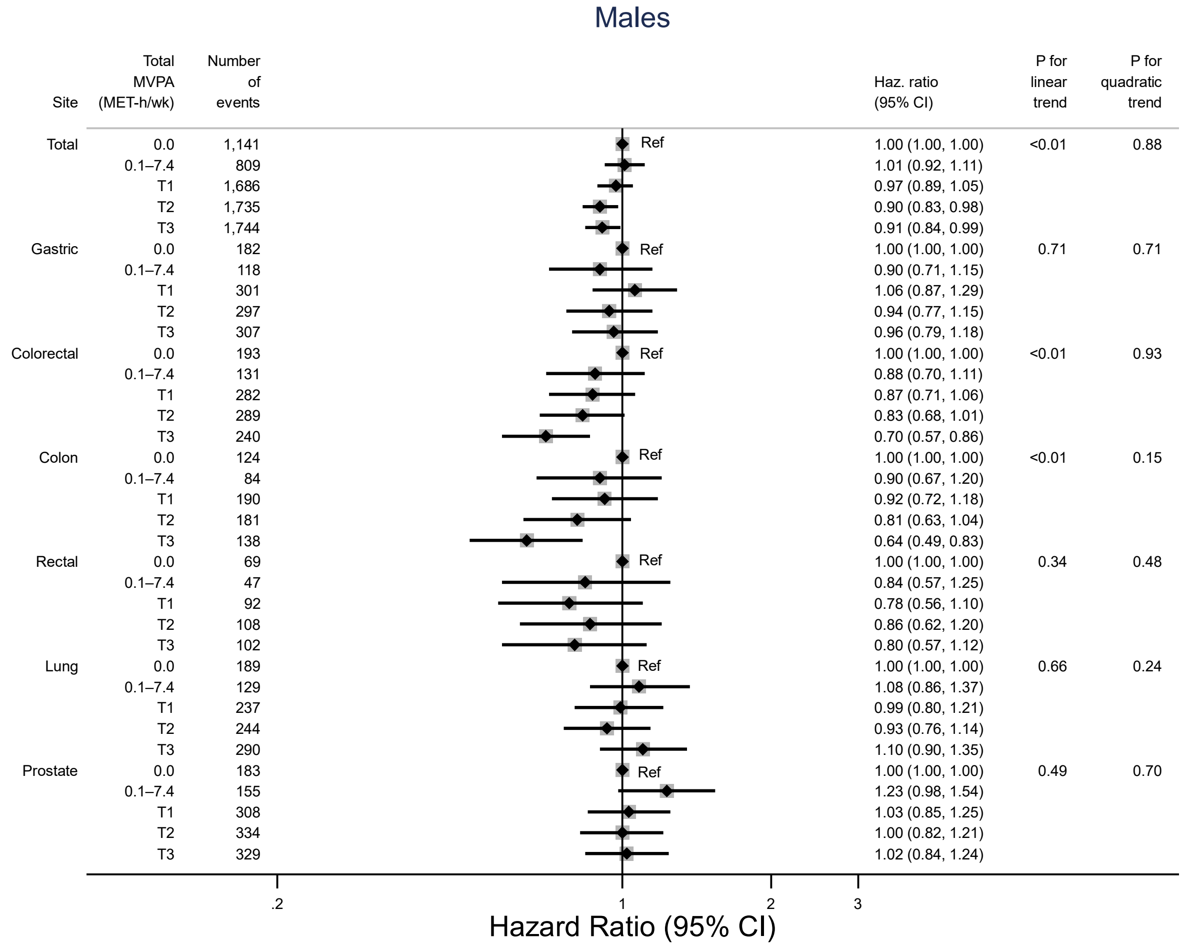

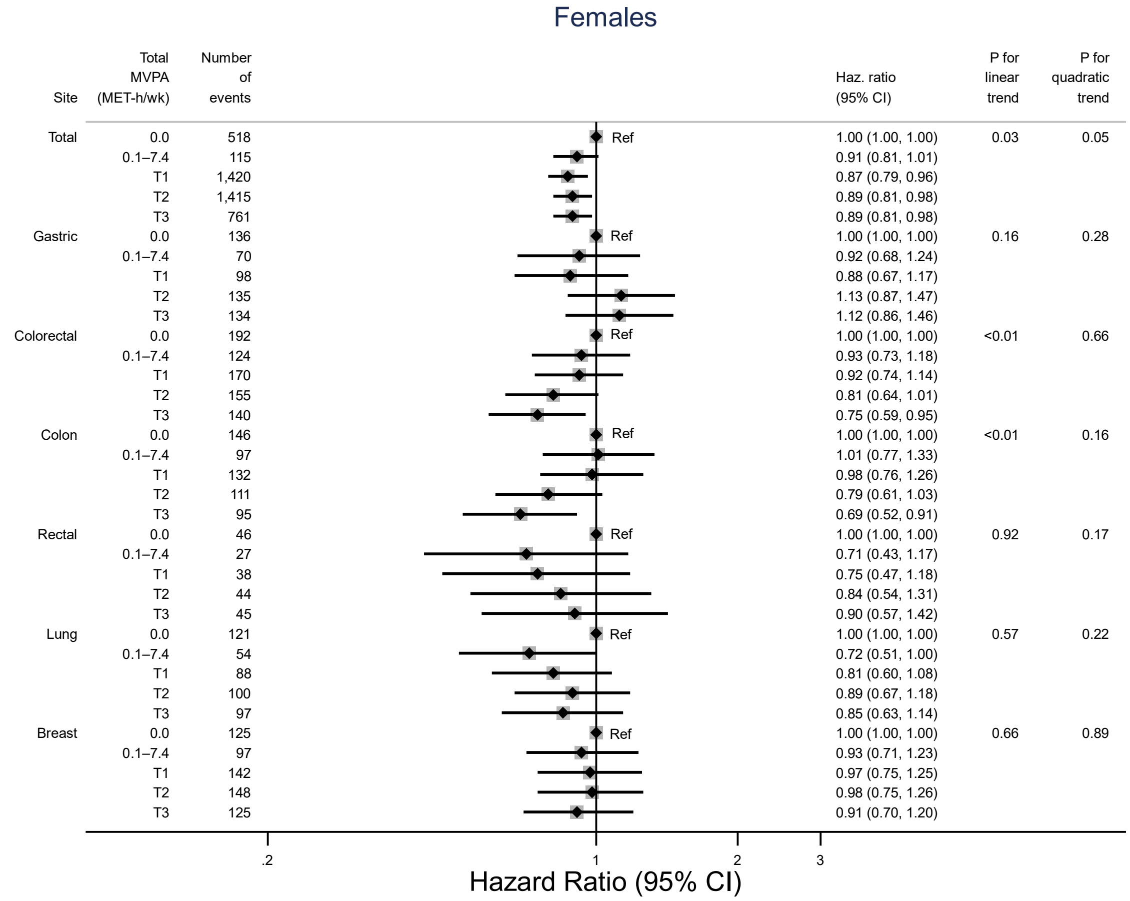

eFigure 5. Associations between categorized amount of MVPA excluding non-leisure-time walking and total and major site-specific cancers.

Cut-off values of total MVPA were calculated in the same way as in Table 1. MVPA calculation excluded non-leisure-time walking. Models were adjusted for the same covariates as those in model 2 of Figure 1 (total cancer) or the model of Figure 2 (site-specific cancers). CI, confidence interval; Haz. Ratio, hazard ratio; MVPA, moderate-vigorous physical activity; T, tertile.

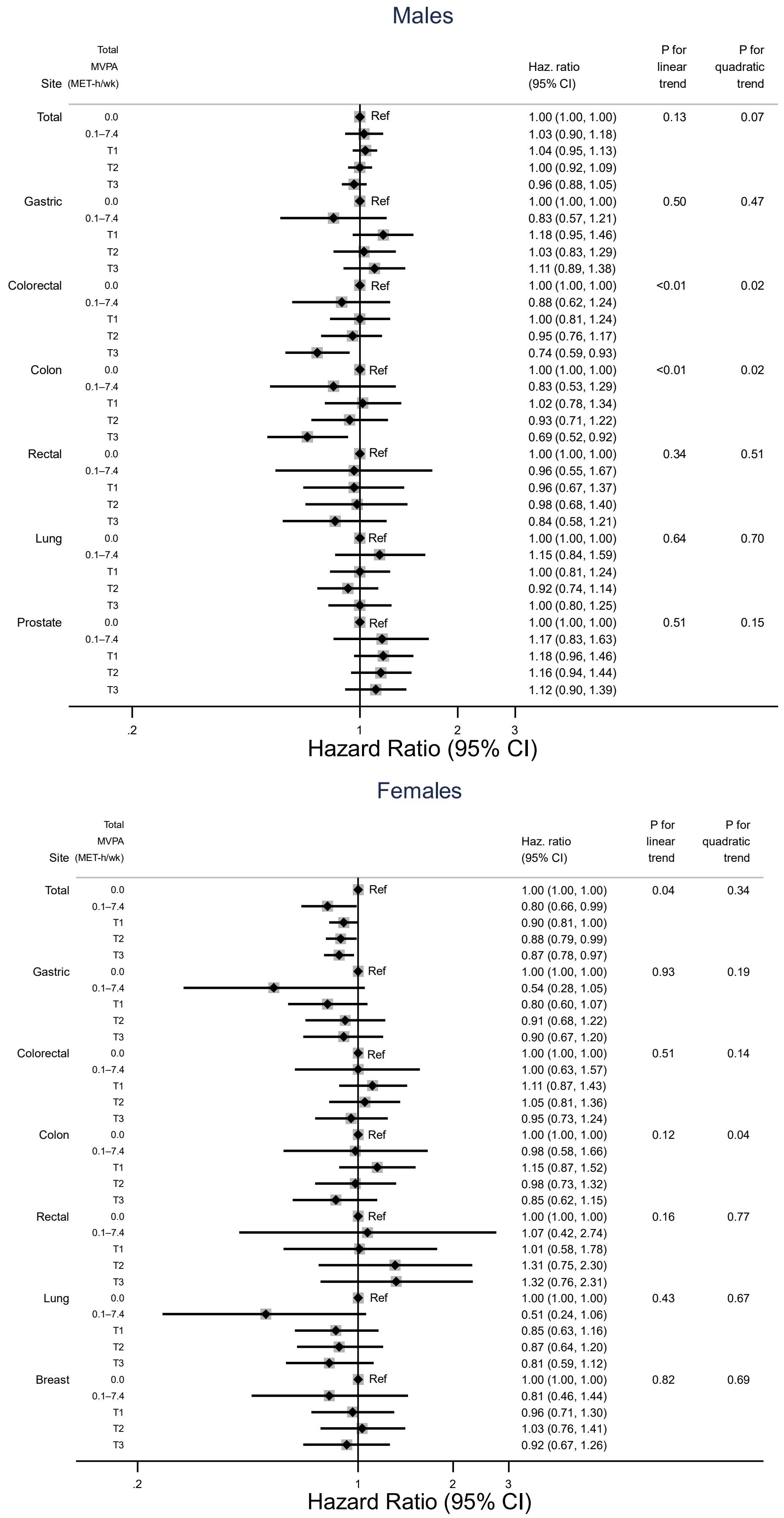

eFigure 6. Associations of categorized amount of total MVPA with overall and major site-specific cancers in each sex, with multiple imputation of missing values.

These analyses included 747 males and 803 females (150 and 92 cases of total cancer) who were excluded from the main analyses due to their missing values in physical activity and imputed missing physical activity and covariates. Cut-off values of total MVPA were the same as in Table 1 or Table 2. Models were adjusted for the same covariates as those in model 2 of Figure 1 (total cancer) or the model of Figure 2 (site-specific cancers). CI, confidence interval; Haz. Ratio, hazard ratio; MVPA, moderate-vigorous physical activity; T, tertile.

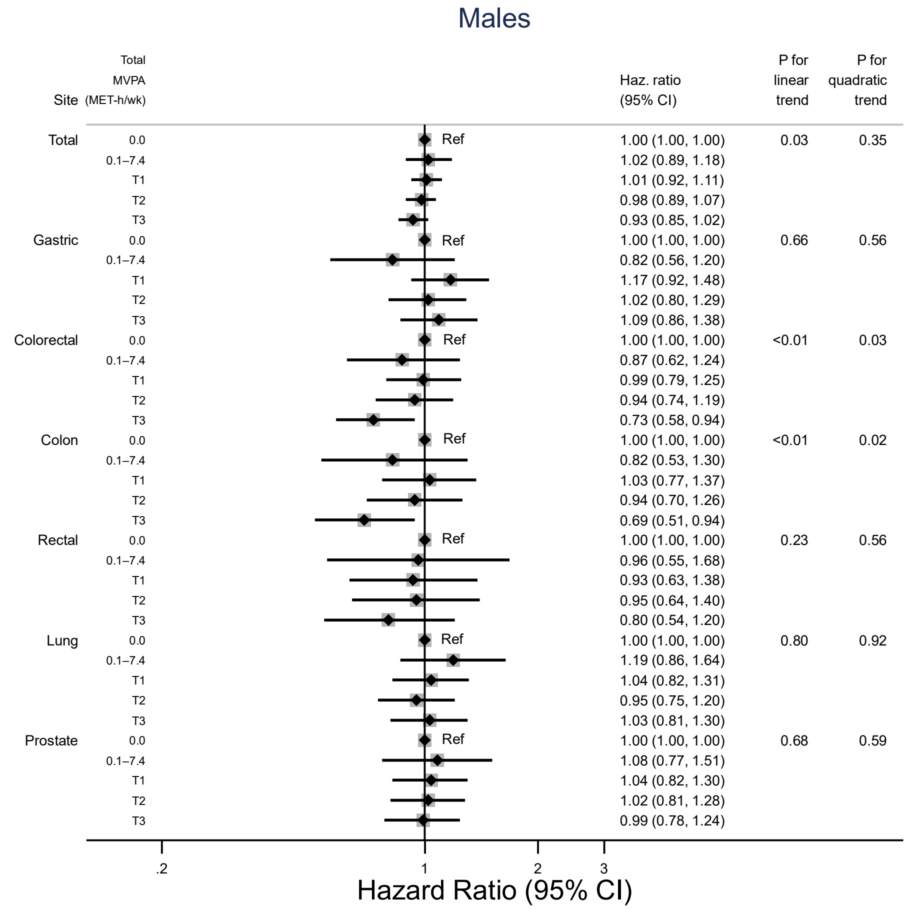

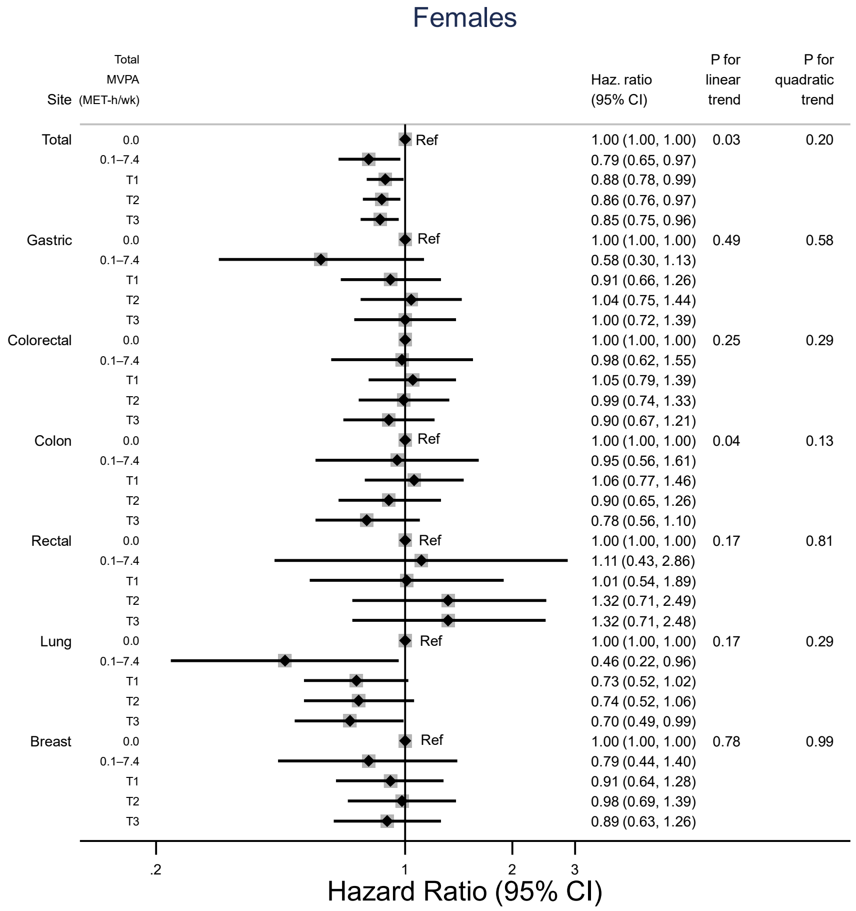

eFigure 7. Associations between categorized amount of MVPA and total and major site-specific cancers with additional covariate adjustment.

Cut-off values of total MVPA were the same as in Table 1 or Table 2. Models were adjusted for use of antihypersentive and cholesterol-lowering drugs, parental history of cancer, age of menarche, and parity (age of menarche and parity were adjusted only in breast cancer) in addition to the covariates in model 2 of Figure 1 (total cancer) or the model of Figure 2 (site-specific cancers). CI, confidence interval; Haz. Ratio, hazard ratio; MVPA, moderate-vigorous physical activity; T, tertile.
